# Infectivity responses of *Salmonella enterica* to bacteriophages on maize seeds and maize sprouts

**DOI:** 10.1016/j.crfs.2024.100708

**Published:** 2024-02-25

**Authors:** Nan Xiang, Catherine W.Y. Wong, Xinbo Guo, Siyun Wang

**Affiliations:** a School of Food Science and Engineering, Guangdong Province Key Laboratory for Green Processing of Natural Products and Product Safety, Engineering Research Center of Starch and Vegetable Protein Processing Ministry of Education, South China University of Technology, Guangzhou, 510640, China; b Food, Nutrition and Health, University of British Columbia, 120-2205 East Mall, Vancouver, BC, V6R 1Z4, Canada

**Keywords:** *Salmonella enterica*, Maize sprouts, Bacteriophage, Gene expression, Seeds, Storage

## Abstract

*Salmonella enterica* (*S. enterica*) is a major foodborne pathogen leading to a large number of outbreaks and bringing food safety concerns to sprouts. The control of *S. enterica* on maize sprouts is important because raw maize sprouts have been gaining attention as a novel superfood. Compared to conventional chemical methods, the applications of bacteriophages are regarded as natural and organic. This study investigated the effects of a 2 h phage cocktail (SF1 and SI1, MOI 1000) soaking on reducing the populations of three *Salmonella enterica* strains: *S.* Enteritidis S5-483, *S.* Typhimurium S5-536, and *S.* Agona PARC5 on maize seeds and during the storage of maize sprouts. The results showed that the phage cocktail treatment effectively reduced populations of *S. enterica* strains by 1–3 log CFU/g on maize seeds and decreased population of *S.* Agona PACR5 by 1.16 log CFU/g on maize sprouts from 7.55 log CFU/g at day 0 of the storage period. On the other hand, the upregulations of flagella gene *pefA* by 1.5-folds and membrane gene *lpxA* by 23-folds in *S.* Typhimurium S5-536 indicated a differential response to the phage cocktail treatment. Conversely, stress response genes *ompR*, *rpoS*, and *recA*, as well as the DNA repair gene *yafD*, were downregulated in *S.* Agona PARC5. This work shows the use of bacteriophages could contribute as a part of hurdle effect to reduce *S. enterica* populations and is beneficial to develop strategies for controlling foodborne pathogens in the production and storage of maize sprouts.

## Highlights

• Phages reduced *Salmonella enterica* populations by 1–3 log CFU/g on maize seeds.
• The phage cocktail reduced *S.* Agona by > 1 log CFU/g on maize sprouts.
• The expression of *lpxA* in *S.* Typhimurium was upregulated by 23 folds.
• The expression of *ompR*, *rpoS*, and *recA* was downregulated by 1–2 folds in *S.* Agona.

## Graphical abstract

**Figure:**
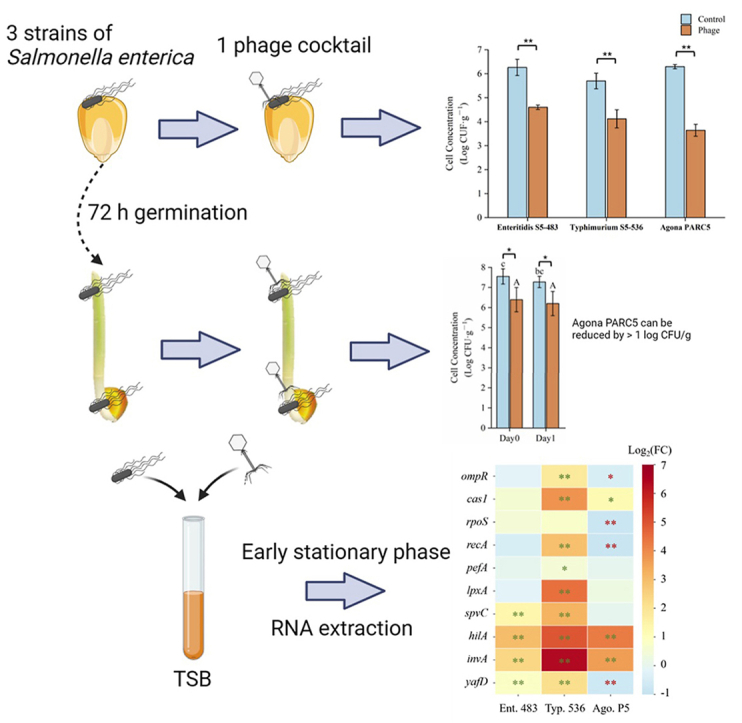

## 1 Introduction

Sprouting is widely recognized as an effective method to enhance the nutritional value and reduce anti-nutrient content of seeds, and the consumption of fresh organic sprouts has substantially increased due to the pursuit of nutritive value (Miyahira et al., 2021; Chen et al., 2022a). Numerous reports over the past decades have highlighted a negative correlation between the consumption of certain sprout products and the risks of various diseases, such as gastrointestinal problems, cardiovascular disorders, and cancer (Aziz et al., 2022). Maize (*Zea mays* L.), a staple food crop cultivated in over 170 countries, boasts a significant yield of approximately 1147.7 million metric tons (Meena et al., 2022). In addition to its high concentration of carbohydrates, dietary fiber, and protein, maize serves as an abundant source of vitamins, amino acids, and minerals (Meena et al., 2022), and provides a large amount of food supply and energy source worldwide (Palacios-Rojas et al., 2020). Germination of maize seeds has been shown to significantly increase the levels of carotenoids, gamma amino butyric acid, total phenolics, total anthocyanins, total flavonoids, and antioxidant capacity, thereby enhancing the nutritional value and health benefits of maize sprouts (Chalorcharoenying et al., 2017; Xiang et al., 2017). Consequently, maize sprouts have gained considerable attention in recent years due to their rich nutrient profile, necessitating further research on production control to facilitate their development as novel food sources to strengthen food security.

However, sprouted vegetables often raise public health concerns due to the risk of foodborne pathogen contaminations (Taormina et al., 1999). Over the past decade, over 10 outbreaks from sprout products were documented, half of which were attributed by *Salmonella enterica* (*S. enterica*) (Control and Prevention, 2023), which can readily contaminate sprout seeds, utilizing the nutrients and moisture available during the germination process, thereby leading to significant food safety issues (Natl Advisory Comm Microbiological, 1999; Miyahira and Antunes, 2021). *S*. e*nterica* is a Gram-negative bacterium that is facultatively anaerobic, flagellated, and has a rod-shaped morphology. It exhibits a low infective dose and is the causative agent of salmonellosis, which manifests as acute enterocolitis accompanied by inflammatory diarrhea (Chen et al., 2022b; Jarvis et al., 2016). Due to the inherent adaptability of pathogenic *S. enterica* and the complex nature of various food environments, controlling its growth and spread in food poses significant challenges (Jarvis et al., 2016). Specifically, seeds or sprouts are exposed to the contamination of *S*. *enterica* during the germination, harvest, and processing stage, primarily through water and fecal matter of animals (Mohle-Boetani et al., 2009; Mahon et al., 1997). Although no outbreaks associated with maize sprouts have been reported thus far, it is essential to consider that because maize sprouts are nutritionally rich, provides abundant carbohydrates, proteins, and amino acids that they can readily support microbial growth. Consequently, there is a crucial necessity to study the growth dynamics and inhibition strategies targeting *S. enterica* in maize sprouts.

Conventionally, sprouts are recommended to be treated with chemical methods including calcium hypochlorite, acetic acid, lactic acid, and hydrogen peroxide, as well as physical methods such as hot water treatment (Food and Regist, 2017). However, researchers have increasingly turned their attention to bacteriophages (phages) as an alternative disinfection method, owing to their high specificity towards pathogens while preserving the native food microbiota (Sillankorva et al., 2012). Previously, specific phages were applied during the sprouting period of alfalfa, mung bean, and lettuce seeds to reduce the population of pathogens such as *Salmonella* and *Escherichia coli* (Ye et al., 2010a; Zhang et al., 2019; Fang et al., 2022; Wong and Wang, 2022). Although phages are stored under strict conditions like refrigerated temperature (typically 2–8 °C) and limited combination with other chemical reagents to avoid being inactivated, they are naturally occurring, self-replicating and are generally nontoxic to humans (Moye et al., 2018). The addition of phages does not introduce any discernible changes in the sensory, textural, or nutritional properties of the food products, indicating itself a promising techniques in food biocontrol (Moye et al., 2018).

Moreover, the antibiotic resistance has grown into a global public health concern, phage therapy to treat or prevent bacterial infections is underwent deeply study worldwide (Labrie et al., 2010). The detailed phage resistance mechanism in host bacteria has been reported including the prevention of phage adsorption, DNA entry, the cleavage of phage nucleic acids, and abortive infection systems (Labrie et al., 2010). In *S. enterica*, the expression of genes related to membrane structure, replication interference, virulence is conducive to the investigation of phage responsive mechanism (Lin et al., 2021). Therefore, effective phages that can target a large host range of *S. enterica* strains were selected (Brenner et al., 2020) for a phage cocktail in the present study to reduce the populations of *S. enterica* strains on maize sprouts throughout the germination and storage stages, meanwhile the underlying mechanisms of different *S. enterica* strain responses were investigated using RT-qPCR analysis.

## 2 Materials and methods

### 2.1 Bacterial strains and growth conditions

Three *S. enterica* strains: *S.* Enteritidis S5-483 and *S.* Typhimurium S5-536, isolated from human, and *S.* Agona PARC5 isolated from alfalfa seeds were individually used in this study. All strains were preserved at −80 °C in tryptic soy broth (TSB, BD, New Jersey, USA) with 20% glycerol (VWR International, Pennsylvania, USA). Working stocks were maintained on tryptic soy agar (TSA, BD, New Jersey, USA) and stored at 4 °C for a maximum of one month. For inoculation, *S. enterica* strains were individually prepared at 37 °C in 3 mL TSB under agitation at 200 rpm for 10 h. One hundred microliter of the resulting suspension were transferred to a new 10 mL TSB and incubated for another 12 h at the same condition. The cultures were then centrifuged at 4000×*g* for 10 min, and the supernatant was decanted. The resulting pellets were washed twice with 10 mL 0.1% peptone water (PW, BD, New Jersey, USA). The final suspension was diluted by sterilized deionized H_2_O (sdH_2_O) to obtain a concentration of ∼10^8^ CFU/mL.

### 2.2 Bacteriophage propagation and plaque assays

Two *Salmonella* phages, SF1 (GenBank: MK770409) and SI1 (GenBank: MK972691), which were previously isolated in British Columbia, Canada, were used in this study (Fong et al., 2017). Both of them are broad host-range phages as classified and are lytic phages with 25 min latency periods. They were characterized free of antimicrobial resistance genes and recombination genes, and were effective in reducing populations of the three studied *Salmonella* strains. *S.* Enteritidis S5-483 strain was utilized for phage propagation. *S. enterica* cultures were prepared by incubating overnight at 37 °C in 10 mL of TSB with agitation at 200 rpm. One hundred microliters of the overnight cultures were then inoculated into 10 mL of TSB and incubated at 37 °C with agitation at 200 rpm for 2 h until the OD600 value reached between 0.2 and 0.4, which was measured using a UV-1800 UV/Vis Spectrometer (Shimadzu, Maryland, USA). Subsequently, 50 μL of phage (SF1 or SI1) was added to the cultures, followed by incubation at 37 °C for 6 h. The mixture was then centrifuged at 4000×*g* for 10 min and the resulting lysate was filtered through a 0.4 μm filter (VWR International, Pennsylvania, USA) and stored at 4 °C until further use.

To assess the efficacy of the phages, a plaque assay was performed for each strain. Each strain was inoculated in 5 mL of TSB at 37 °C with agitation at 200 rpm for 18 h. Thirty microliters of the cultures were diluted with 270 μL of TSB and mixed with 4 mL of 0.7% TSA (pre-heated to 55 °C). The mixture was immediately poured onto a TSA plate to form a smooth agar layer. Then, the phage lysate was diluted accordingly with SM buffer (Thermo Scientific Chemicals, Massachusetts, USA) to different concentrations which were then separately added on the surface of the TSA as droplet about 5 μL. The plates were incubated at 37 °C for 24 h prior to plaque counting.

### 2.3 Inoculation of *S. enterica* strains on maize seeds

Maize seeds were purchased from Amazon (Food to Live, New York, USA). Thirty grams of maize seeds were weighted and treated with 70% ethanol for 5 min under agitation at 200 rpm in glass bottle. Then, ethanol was decanted and seeds were washed by sdH_2_O for ten times to thoroughly remove the ethanol. The sterilized maize seeds were soaked in sdH_2_O in sterilized tip boxes (122*87*65 mm) for 12 h. After imbibition, maize seeds were dried on filter paper. Subsequently, they were treated with different *S. enterica* strains obtained in section 2.1 in the tip boxes at the proportion of 5 g/mL according to group. Tip boxes were shaken by hand for 2 min to achieve homogenous *S. enterica* inoculation on the seeds. Samples were air-dried in biosafety cabinet for 1 h, with a 30 s shake every 15 min, resulting in a final *S. enterica* concentration of ∼10^6^ CFU/g on seeds.

To determine the microbiological quality of maize seeds, 20 g of maize seeds (before disinfection) were weighted and added with 20 mL 0.1% PW in Whirl Pak bags (Nasco Whirl-Pak, Madison, USA) for estimation of total aerobic population and *S. enterica* populations on TSA and xylose lysine deoxycholate (XLD) agar (Oxoid, Ontario, Canada), respectively.

### 2.4 Antimicrobial treatment on maize seeds

Maize seeds separately inoculated with *S. enterica* strains were soaked with the phage cocktail (SF1 + SI1) at MOI 1000 with agitation at 200 rpm for 2 h (phage group) according to previously reported study (Wong and Wang, 2022). After 2 h, the liquids were decanted and the seeds were averagely placed in tip boxes. Twelve millimeters of sdH_2_O were added in each box for maize seeds germination. The positive control group were comprised of maize seeds that were inoculated with the *S. enterica* strain and directly placed into the tip boxes for germination without further treatment. The maize seeds without any treatment were set as the negative control group. Two grams of maize seeds were taken out and dried on filter paper, added with 8 mL of 0.1% PW in Whirl Pak bags, and vortexed for 2 min. The mixture was diluted for population estimate of *S. enterica* on XLD agar. Each treatment was conducted with three biological replicates. All seeds were then cultivated in tip boxes in the dark at 28 ± 1 °C for 72 h.

To determine the germination rate of maize seeds in each group, 10 g of maize seeds were inoculated with *S. enterica* strains as mentioned in section 2.3 and inoculated as described in section 2.4. The germination rate of maize seeds from all treatments were calculated after 24 h of germination according to the formula: Germinationrate(%)=Numberofgerminatedseeds÷Numberoftotalseeds×100%.

### 2.5 Antimicrobial treatment on maize sprouts

After 72 h of germination, the maize sprouts in the positive control group were used for the storage experiment. Subsequently, sprouts inoculated with *S. enterica* strains were separately treated with phage cocktail (SF1 + SI1) at MOI 1000 with agitation at 200 rpm for 2 h. Liquids were decanted and maize sprouts were then placed in food preservation boxes (S.C. Johnson, Wisconsin, USA) for 10 days at 8 °C. After 0, 1, 2, 3, 6, and 10 days, 2 g of maize sprouts were added with 8 mL of 0.1% PW in Whirl Pak bags and vortexed for 2 min. The mixture was diluted in 0.1% PW to estimate the population of *S. enterica* on XLD agar.

### 2.6 Microbiological analyses of *S. enterica* strains

*S. enterica* strains were individually prepared in 10 mL TSB at 37 °C with agitation at 200 rpm overnight. Then, one hundred microliter suspension were transferred to a new 10 mL TSB and incubated at 37 °C with agitation at 200 rpm for another 16 h to achieve a maximum population of ∼10^9^ CFU/mL. Further, suspension was diluted to ∼10^8^ CFU/mL. Part of the diluted suspension was then incubated with agitation at 200 rpm for 10 h at 37 °C as the control group. For the other part of the diluted suspension, it was added with the phage cocktail (SF1 + SI1) at MOI 1000 and incubated at 37 °C with agitation at 200 rpm for 10 h as the phage group. A one hundred microliter suspension was then diluted accordingly for plating on XLD agar at 0 min, 1 min, 30 min, 1 h, 2 h, 4 h, 6 h, 8 h, and 10 h. This protocol was repeated independently for each strain in three replicates.

### 2.7 RNA extraction, reverse-transcription, and real time quantitative PCR (RT-qPCR)

Cultures of each *S. enterica* strain (from section 2.6) in the early stationary phase of each group were used for RNA extraction. To ensure the stability of the RNA, the cultures were first treated with RNAprotect Bacteria reagent (Qiagen, Hilden, Germany). The RNA extraction was performed using the RNeasy Mini Kit (Qiagen, North Rhine-Westphalia, Germany) with on-column DNase digestion (Qiagen, North Rhine-Westphalia, Germany). Three replicates of RNA extractions were carried out for each group. The quantity and quality of the extracted RNA were assessed using a Nanodrop Spectrophotometer (Thermo Scientific Inc., North Carolina, USA) and an Agilent 2100 Bioanalyzer system (Agilent, California, USA). Subsequently, the extracted RNA was reverse-transcribed using the QuantiTect Reverse Transcription Kit (Qiagen, North Rhine-Westphalia, Germany) in a Veriti 96 Well Thermal Cycler (Applied Biosystems, Massachusetts, USA). The resulting cDNA was stored at −20 °C until further analysis. For RT-qPCR, the Quantinova SYBR Green PCR kit (Qiagen, North Rhine-Westphalia, Germany) was used in a C1000 Touch Thermal Cycler (BIO-RAD, California, USA). The 16S rRNA gene was used as the reference gene with forward primer: 5′-CGGGGAGGAAGGTGTTGTG-3′ and reverse primer: 5′-GAGCCCGGGGATTTCACATC-3′ (Dong et al., 2021). The primers used for the target genes are listed in Table 1. The relative expression values of the genes were calculated using the 2^−ΔΔCt^ method. The results are presented as mean ± SD (n = 3).

**Table 1:** Primers used for the target genes.

Gene name	Gene description	Primer direction	Sequence (5’→3′)
*ompR*	two-component system response regulator OmpR (Lin et al., 2021)	Forward	GTGAAGATGAACCGATGCCG
		Reverse	GCCGGATCTTCTTCCACCAT
*cas1*	CRISPR/Cas system-associated protein Cas1 (Wang et al., 2019)	Forward	GCAAAGCTGGCGTTAGATGA
		Reverse	GATCCTTCAATACCGCGCAG
*rpoS*	RNA polymerase sigma factor rpoS (Yang et al., 2014)	Forward	GAATCTGACGAACACGCTCA
		Reverse	CCACGCAAGATGACGATATG
*recA*	recombination protein RecA (Wang et al., 2019)	Forward	GATATCCGTCGTATTGGCGC
		Reverse	CCGTTGTAGCTGTACCATGC
*pefA*	plasmid-encoded fimbriae; major fimbrial subunit (Lin et al., 2021)	Forward	GCGTGAACTCCAAAAACCCG
		Reverse	TTGAAGTCACCTTCGGTCGC
*lpxA*	UDP-N-acetylglucosamine acyltransferase (Wang et al., 2019)	Forward	AAGCGTCACCATTCATCGTG
		Reverse	GATGAACTGCCGTCATACCG
*spvC*	*Salmonella* plasmid virulence protein (Mazurkiewicz et al., 2008)	Forward	ATTTGCCGGTGACAAGTTCC
		Reverse	GGAGAAACGACGCACTGTAC
*hilA*	transcriptional regulator hilA (Wang et al., 2019)	Forward	ATTAAGGCGACAGAGCTGGA
		Reverse	GCAGAAATGGGCGAAAGTAA
*invA*	attachment/invasion protein (Ye et al., 2018)	Forward	ACCGTGGTCCAGTTTATCGT
		Reverse	GCTTTCCCTTTCCAGTACGC
*yafD*	putative cytoplasmic protein (Lin et al., 2021)	Forward	ATTTTAGTCTGGGCGTGGAC
		Reverse	AACAAAATCCAGCGGTCGTC

### 2.8 Statistical analyses

Each experiment was conducted with three biological replicates. One-way ANOVA was conducted to analyze the significant differences among samples and Ducan's comparison post-tests were used for means separation by using IBM SPSS Statistics 25.0 (SPSS Inc., Chicago, USA) software (p < 0.05). T-tests were applied between groups to determine the significant differences with MetaboAnalyst 5.0 (Pang et al., 2021). Figures were depicted on Origin 2018 (OriginLab Corporation, Northampton, USA) with modifications. All results were expressed as mean ± SD (n = 3).

## 3 Results

### 3.1 Microbiological quality of maize seeds

The maize seeds employed in this study exhibited an estimated total aerobic population of 3.24 ± 0.03 log CFU/g. Subsequently, the absence of *S. enterica* was observed following a 24-h incubation period at 37 °C.

### 3.2 The germination rate of maize seeds

The germination rates of maize seeds across various treatment groups are listed in Table 2. The maize seeds exhibited a notably high germination rate of 97.22%. However, upon exposure to *S. enterica*, the germination rate decreased to a range of 75–79%. The application of a phage cocktail to seeds contaminated with *S. enterica* had no influences on the germination rate of maize seeds, as the germination rate ranged from 74 to 77 %. Similarly, the reducing germination rate of maize seeds was found in a previous study when *S.* Typhimurium was inoculated onto the seeds (Singh et al., 2004).

**Table 2:** The germination rate of maize seeds.

Germination rate (%)		Positive Control	Phage cocktail
Negative Control	Strains		
97.22 ± 4.81a	*S. enterica* Enteritidis S5-483	77.64 ± 8.68b	75.62 ± 5.58b
	*S. enterica* Typhimurium S5-536	78.75 ± 6.58b	74.44 ± 3.85b
	*S. enterica* Agona PARC	75.38 ± 5.30b	76.54 ± 10.69b

Different letters (a-b) depicted for significant differences (p < 0.05).

### 3.3 The varied concentration of *S. enterica* on maize seeds after phage treatment

**Fig. 1:**
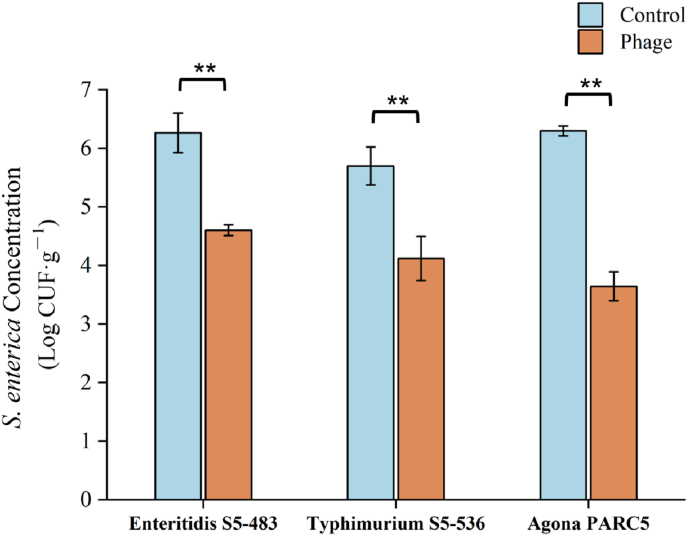
The reduction of *S. enterica* strains on maize seeds. T-test were conducted between control and phage groups. **: p < 0.01.

According to Fig. 1, the concentration of *S. enterica* on maize seeds within the control groups was approximately 6 log CFU/g. Among the three strains of *S. enterica* inoculated onto the maize seeds, *S.* Agona PARC5 was the most susceptible to the phage cocktail treatment, resulting in a reduction of approximately 2.7 log CFU/g (p < 0.01). Moreover, the application of the phage cocktail yielded reductions of approximately 1.7 log CFU/g and 1.6 log CFU/g for *S.* Enteritidis S5-483 and *S.* Typhimurium S5-536 strains, respectively. In general, the utilized phage cocktail exhibited effectiveness against all three selected strains, resulting in reductions exceeding 1 log CFU/g.

### 3.4 The varied concentration of *S. enterica* during sprouts storage

**Fig. 2:**
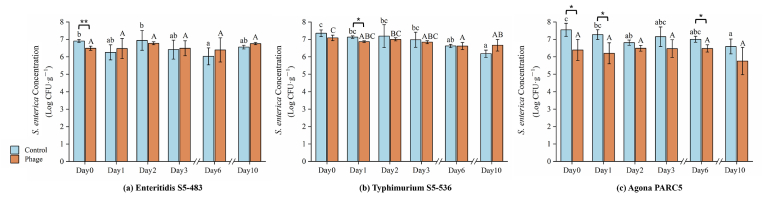
The concentration of *S. enterica* strains during maize sprouts storage. T-test was operated between control and phage group. *: p < 0.05; **: p < 0.01. Different letters (a-b and A-B) in each column depict significant differences within control and phage groups.

Fig. 2 illustrates the effects of phage cocktail treatment on the populations of *S.* Enteritidis S5-483, *S.* Agona PARC5, and *S.* Typhimurium S5-536 on maize sprouts during ten days storage period at 8 °C. Specifically, on day 0, the phage cocktail treatment led to a notable reduction in the populations of *S.* Enteritidis S5-483 and *S.* Agona PARC5. More precisely, the treatment resulted in a reduction of 0.42 ± 0.02 log CFU/g in the population of *S.* Enteritidis S5-483 (p < 0.01), while displaying greater effectiveness against *S.* Agona PARC5 with a reduction of 1.16 ± 0.24 log CFU/g (p < 0.05). In contrast, the phage cocktail treatment did not significantly alter the density of *S.* Typhimurium S5-536 on maize sprouts.

Overall, the concentrations of *S. enterica* in the control groups of the three strains showed a decreasing trend. The lowest concentrations of *S.* Enteritidis S5-483, *S.* Typhimurium S5-536, and *S.* Agona PARC5 were recorded on day 6, day 10, and day 10, respectively, with values of 6.02 ± 0.49, 6.18 ± 0.21, and 6.59 ± 0.43 log CFU/g. Conversely, the phage cocktail treatment did not affect the concentrations of *S.* Enteritidis S5-483 and *S.* Agona PARC5 during the storage period, whereas a reduction in the concentration of *S.* Typhimurium S5-536 was observed after the 10-day storage (p < 0.05).

The maize sprouts utilized for storage underwent a 3-day germination period during which they were exposed to *S. enterica*, and it was observed at day 0 that *S.* Agona PARC5 exhibited the highest susceptibility to the phage cocktail treatment, followed by *S.* Enteritidis S5-483 (Fig. 2). Furthermore, the storage of maize sprouts at low temperatures proved effective in inhibiting the growth of *S. enterica* and reducing the potential risks associated with pathogens.

### 3.5 The recovery curves of *S. enterica* in TSB

**Fig. 3:**
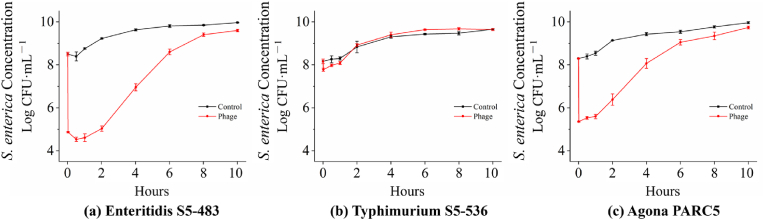
The recovery curves of *S. enterica* strains in TSB.

The phage cocktail treatment was applied to cultures inoculated with three strains of *S. enterica* separately, and the concentrations were monitored at different time points, as illustrated in Fig. 3. In the control group, *S.* Enteritidis S5-483 populations gradually reached a stationary phase by 4 h. For the phage treatment group, the concentration of *S.* Enteritidis S5-483 immediately decreased from approximately 8 log CFU/mL to 5 log CFU/mL, with a significant reduction of 3.63 ± 0.09 log CFU/mL. Subsequently, there was a continuous downward trend in population for approximately 30 min. However, *S.* Enteritidis S5-483 began to recover and demonstrated population resurgence between 30 min and 1 h. From 1 to 8 h, the population steadily increased to 9.40 ± 0.08 log CFU/mL, entering the early stationary phase.

Regarding *S.* Typhimurium S5-536, it followed a similar pattern, taking 4 h to reach stationary phase for the control group. However, when treated with the phage cocktail, only a reduction of 0.36 ± 0.12 log CFU/mL was observed. Between 1 min and 2 h, the *S. enterica* exhibited a rapid increase, reaching the same level as the control group. Subsequently, the *S. enterica* population slightly increased to 9.40 ± 0.12 log CFU/mL, indicating the onset of the early stationary phase. From 4 to 10 h, both the control and phage cocktail groups maintained the concentrations of approximately 9 log CFU/mL, signifying the stationary phase.

Among the three strains, *S.* Agona PARC5 demonstrated the highest sensitivity to the phage cocktail treatment in TSB. In the control group, the population reached approximately 9 log CFU/mL within 4 h. However, with the phage cocktail, the concentration significantly dropped to 5.35 ± 0.02 log CFU/mL. Unlike *S.* Enteritidis S5-483, *S.* Agona PARC5 exhibited population growth between 1 min and 1 h. Subsequently, the concentration dramatically increased to 9.05 ± 0.13 log CFU/mL at 6 h, indicating the early stationary phase.

Overall, *S.* Enteritidis S5-483 was the most susceptible strain to the phage cocktail in TSB, with an inhibition of over 3 log CFU/mL occurring within 1 min. On the other hand, *S.* Typhimurium S5-536 demonstrated resistance to the phage cocktail. These resistance patterns observed among the three strains in TSB were consistent with those observed on maize seeds and sprouts.

### 3.6 Transcriptional response of *S. enterica* to phage treatment

**Fig. 4:**
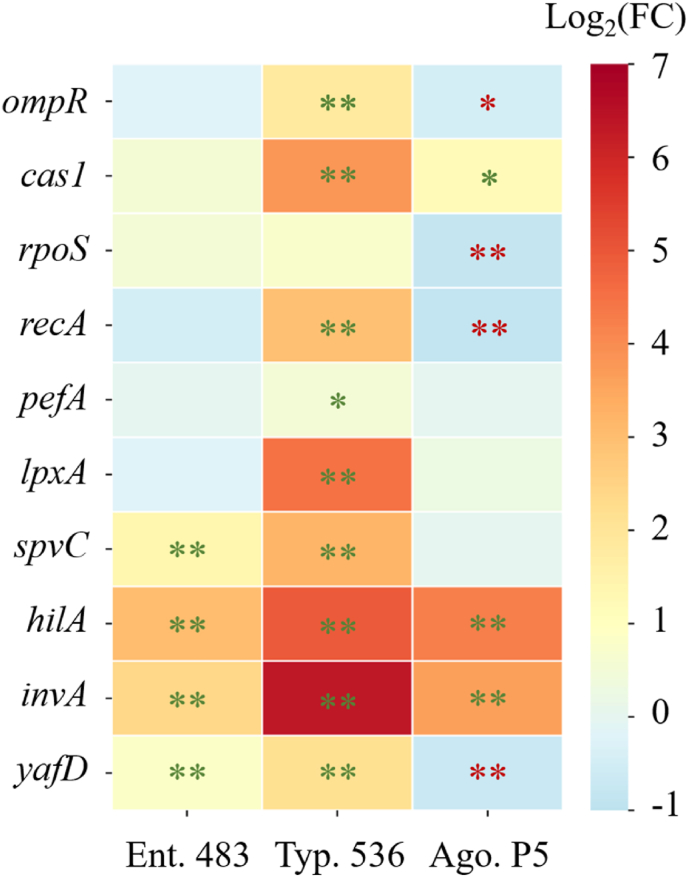
The Log_2_(FC) values of genes (Phage group *vs* Control group) in *S. enterica* strains at early stationary phase. T-test was operated between control and phage group. *: p < 0.05; **: p < 0.01. The green marks stand for the upregulations by phage treatment while the red marks stand for the downregulations.

Bacterial resistance and gene expression to various lethal conditions are highly dependent on the growth phase, hence recover modeling was conducted in this study to ensure that cells in the same early stationary phase were used to evaluate relative genes and the results are shown in Fig. 4. A total of ten genes were studied, including the stress response genes *ompR*, *cas1*, *rpoS*, and *recA*, the genes associated with the integrity and morphology of the flagella and cell membrane *pefA* and *lpxA*, the virulence genes *spvC*, *hilA*, and *invA*, as well as *yafD*, which is involved in DNA repair.

The results showed that the three virulence genes and *yafD* of *S.* Enteritidis S5-483 were significantly upregulated in response to phage cocktail treatment after 8 h (p < 0.01). In contrast, the expression of other genes remained similar to the control group. In the case of *S.* Typhimurium S5-536, most of the detected genes exhibited upregulation. Notably, the expression of *invA* gene showed a remarkable increase after treated by the phage cocktail. Gene *hilA* was also significantly upregulated by 31-fold as compared to the control group. Moreover, the expressions of membrane gene *lpxA* and flagella gene *pefA* were increased by 23- and 1.5-folds, respectively. The stress response genes in *S.* Agona PARC5 exhibited diverse expression patterns upon the phage cocktail treatment, with *ompR*, *rpoS*, and *recA* being inhibited, while *cas1* was upregulated. Two virulence genes were upregulated, while the DNA repair-related gene was downregulated. Overall, the expression of virulence genes demonstrated consistent trends among the three selected strains, while the expression of other genes specifically varied in response to the phage cocktail treatment, depending on the strains.

## 4 Discussion

### 4.1 The effectiveness of the phage cocktail to reduce *S. enterica* on maize seeds and sprouts is strain-dependent

The phage cocktail used in this study consisted of two effective phages with wide host ranges (Brenner et al., 2020) and achieved reduction of 1–3 log CFU/g on *S.* Enteritidis 483, *S.* Typhimurium 536 and *S.* Agona PARC5. The findings were similar to a previous study where a single phage, Phage-A was applied on mustard seeds and a 1.37 log suppression of *Salmonella* growth was achieved (Pao et al., 2004). In another study, phage application also demonstrated a strong anti-*Salmonella* Typhimurium effect in 1.8 log reduction on contaminated mung bean seeds after overnight storage at 22 °C (Liao et al., 2022). Ye et al. (2010) used a phage cocktail and achieved a higher *Salmonella* population reduction of ∼3.41 log CFU/g (Ye et al., 2010b). Furthermore, repeating phage cocktail treatment on alfalfa sprout seeds was found to be more effective on inhibiting the recovery of *S. enterica* (Wong and Wang, 2022). Aside from phage treatments, the reduction of *S. enterica* on seeds has also been widely studied by using chemical reagents. Previously, a 5-log reduction in *Salmonella* populations were detected in contaminated alfalfa seeds by applying 20,000 ppm Ca(OCl)_2_ for 10 min (Gandhi and Matthews, 2003). The initial populations of *S. enterica* (6.3–6.5 log CFU/g) on cabbage and chili pepper seeds were treated by 3000 ppm ClO_2_ gas for 10 min and were reduced by > 5.3 log CFU/g (Li et al., 2022). Although superior in removing pathogens, chemical reagents like chlorine are corrosive and can damage food processing equipment (Moye et al., 2018). Moreover, chemical reagents kill microbes indiscriminately, therefore, in order to minimize the effects of treatment on sprouts’ quality and develop a targeted antimicrobial approach, phages have gained popularity for the use in pathogen inactivation (Moye et al., 2018; Endersen and Coffey, 2020).

It is worth noting that *Salmonella* survivors were detected after phage treatments, however, the inoculated *Salmonella* concentrations in this and previous studies could be higher than contamination levels in a food plant. The high inoculated level is a typical method in food microbiology for challenge studies, but in reality, the pathogen contamination can be much lower. At lower *Salmonella* contamination levels, phages could potentially eliminate pathogen levels. To the best of our knowledge, this is the first time that the reductions of *S. enterica* populations on maize seeds and sprouts has been reported, which provides an option as a food safety method for the novel plant sprouts food.

On the other hand, it has been reported that although approximately 1 log CFU/g reduction of viable *Salmonella* could be achieved 3 h after phage application on alfalfa seeds, the phage had no inhibitory effect on *Salmonella* population growth thereafter (Kocharunchitt et al., 2009). The additional second application of phage failed to reduce the population of *Salmonella* on contaminated alfalfa seeds either (Kocharunchitt et al., 2009). On mung bean seeds, although a reduction of *Salmonella* was observed after treatment with SalmoFresh (a *Salmonella* phage cocktail), *Salmonella* was able to grow exponentially during germination (Zhang et al., 2019). By using laser scanning confocal microscopy, Wong et al. (2019) found that the *Salmonella* inoculated on tomato and lettuce seedlings lettuce after 5 days were primarily located inside stomata or in surface depressions adjacent to stomata for survival, indicating the challenge to remove bacteria after inoculating for a period. In a previous study, it was shown that *Salmonella* population was hard to be decreased after seeds were contaminated for 3 h (Kocharunchitt et al., 2009). Considering such situation, most of the approaches of reducing *Salmonella* populations by using phages often involve applying the phages in advance or within one day of *Salmonella* contamination of the plant food. For example, a previous study demonstrated >3 log CFU/g reductions on populations of *S.* Enteritidis strain S3, *S.* Javiana S203, and *S.* Javiana S200 on both lettuce and cantaloupe tissues with phage treatment, which was applied 24 h prior to inoculation with *S. enterica* (Wong et al., 2020). Spot-inoculation of *Salmonella* was subsequently followed by the application of SalmoFresh after 3 h and the phage cocktail was able to decrease *Salmonella* populations on lettuce and mung bean sprouts (Zhang et al., 2019). Moreover, green tea extract is a common neutralizer used for phage neutralization, mostly due to its high catechin content (Dhowlaghar and Denes, 2023; Marongiu et al., 2021). Nevertheless, sprouts are rich in catechin, too so they serve as a natural neutralizer for phages (Francis et al., 2022). As a result, the efficacy of phages is not that prominent on sprouts compared to some other food products. However, the application of phage cocktail in the present study was able to decrease *S.* Agona PARC5 population by more than 1 log CFU/g on maize sprouts inoculated with the bacteria for 72 h, indicating a different resistance mechanism of this strain, which will be discussed with the gene expression results from the present work.

### 4.2 The transcriptional response of *S. enterica* against the phage cocktail is strain-specific

Phages are viruses that can kill specific target bacterial strains, thus can be applied in food, medical and the environment for biocontrol of pathogenic bacteria. However, phage resistance of bacteria is one of the obstacles to phage application, including the inhibition of phage attachment, cleavage of the invading phage genome, replication interference exerted by bacterial CRISPR/Cas systems and the inductive abortion of phage infection (Labrie et al., 2010). To interfere with the replication of phage, gene *cas1*, encoding CRISPR/Cas system-associated protein Cas1, targets the invasive nucleic acid in the host cell for degradation (Silas et al., 2016). Additionally, the *recA* gene encodes for the RecA protein, which is able to promote DNA repair and cell survival in the face of DNA damage by regulating the SOS response pathway in bacteria (Pourahmad Jaktaji and Pasand, 2016). The relative gene expression level of *recA* was increased in *S. enterica* strains by phage, which enables cell survival in the presence of extensive DNA damage (Wang et al., 2019). Moreover, when encountering phage attacks, certain stress-related genes were involved and played an important role in the resistance mechanism. The specific sigma factors σS (encoded by *rpoS* gene) was able to initiate stress-related genes and support the survival of *Salmonella* spp. in stationary phase and under environmental changes (Yang et al., 2014). The OmpR drives acid and osmotic stress responses in single bacterial cells in *S.* Typhimurium, indicating the indispensable role of *ompR* gene in stress response (Chakraborty et al., 2017). On the other hand, OmpR is functional to activate the expression of the *csgD* gene, thereby promoting the expression of the rdar morphotype, which is a bacterial phenotype characterized by the expression of curli fimbriae and cellulose (Römling et al., 2003). Both the *rpoS* and *ompR* genes have been previously observed to be upregulated under the stressful conditions (Chakraborty et al., 2017). In the present findings, the upregulations of the aforementioned genes in *S.* Typhimurium S5-536 indicated the initiation of a stress response, likely contributing to its high resistance against phage treatment. In contrast, phage treatment led to a decrease in the relative expression levels of *ompR*, *rpoS*, and *recA* in *S.* Agona PARC5, accompanied by a diminished resistance of this strain, resulted in the highest decrease in population compared to the other three strains examined.

Lipopolysaccharide (LPS) plays a critical role in protecting bacteria from environmental conditions and signaling pathways (Zhang et al., 2013). As a major structural constituent integrated into the outer membrane of gram-negative bacteria, LPS maintains bacterial membrane integrity, prevents environmental stress, and contributes to antibiotic resistance (Wang et al., 2020). In *Salmonella*, LpxA is essential for lipid A synthesis, a key component of LPS (Wang et al., 2020). In the present results, the upregulation of *lpxA* gene was observed in *S.* Typhimurium S5-536 following phage cocktail treatment, indicating an increase in LPS synthesis. Additionally, the *pefA* gene, which encodes the serotype associated plasmid (SAP) and is involved in fimbrial major subunit antigen production in *Salmonella* Typhimurium (Woodward et al., 1996), was specifically detected and upregulated in response to phage treatment in the current results. The upregulation of *pefA* is known to initiate biofilm formation and is associated with stress response in *Salmonella* Hessarek (Lin et al., 2021). Hence, the observed upregulation of these cell structure-related genes demonstrates the enhanced resistance of *S.* Typhimurium S5-536 against phage interaction.

The relative expression levels of detected virulence genes in three *S. enterica* strains were found to be increased in the phage-treated groups. The *spvC* gene known to be a type III secretion system (T3SS) effector, is closely related to bacterial adhesion, colonization, and serum resistance factors (Wang et al., 2019). For the *hilA* gene, which belongs to *Salmonella* Pathogenicity Island 1 (SPI-1), encodes a transcriptional regulator essential for *Salmonella* spp. invasion during infection (Yang et al., 2014). The *invA* gene, also part of SPI-1, is essential for *Salmonella* to enter cultured epithelial cells (Ye et al., 2018). As reported, high temperature stress induced upregulations of both *spvC* and *hilA* in *Salmonella* (Yang et al., 2014; Valone et al., 1993). The enhanced virulence and biofilm formation of *S.* Typhimurium by phage PHB48 can improve its colonization and contamination ability in food samples (Wang et al., 2023). However, the virulence gene *hilD* expression reduced the resistance of *S*. Typhimurium to outer membrane disrupting treatments, indicating a substantial fitness cost to *Salmonella* (Sobota et al., 2022). Therefore, while the upregulations of virulence genes in the present study implies an elevated potential for *Salmonella* contamination, it is concurrently indicative of reduced stress resistance abilities, consequently prompting the development of anti-virulence strategies.

The studied *yafD* gene is homologous to members of an exonuclease-endonuclease-phosphatase family, including some enzymes involved in DNA repair (Lu et al., 2003). It was significantly upregulated under stress condition and provided the survivability of *Salmonella* Hessarek in egg contents (Lin et al., 2021). Therefore, both *S.* Enteritidis S5-483 and *S.* Typhimurium S5-536 demonstrated the ability to survive phage infection, whereas a downregulated *yafD* gene after phage treatment was detected in *S.* Agona PARC5, indicating its impaired survivability which made itself be more easily eliminated from maize seeds, particularly on maize sprouts.

Overall, the research findings revealed that *S.* Enteritidis S5-483, despite being the most sensitive strain towards phage treatment in TSB, exhibited a recovery period during which its gene expression levels returned to a similar level compared to the control group. The relative gene expressional values of *S.* Typhimurium S5-536 exhibited the most significant change among the three strains, suggesting its high resistance ability. On the other hand, *S.* Agona PARC5 displayed downregulation of certain genes, particularly related to stress response and DNA repair, implying a potentially lower ability of this strain to withstand phage-induced stress, Therefore, among the three strains, despite exhibiting intermediate sensitivity in TSB, *S.* Agona PARC5 was the most susceptible to phage treatment on maize seeds and sprouts.

## 5 Conclusion

In summary, the phage cocktail treatment reduced the population of *S. enterica* strains on maize seeds by 1–3 log CFU/g. On maize sprouts, phages reduced *S.* Agona PACR5 by more than 1 log CFU/g, even after 72 h of contamination. The upregulation of flagella gene *pefA* and membrane gene *lpxA* in *S.* Typhimurium S5-536 indicated resistance to phage treatment, while the downregulations of *ompR*, *rpoS*, and *recA* in *S.* Agona PARC5 suggested the susceptibility of this strain towards the applied phage cocktail. Based on the present work, a broader range of *Salmonella enterica* strains is needed to provide a more comprehensive understanding of phage efficacy. Additional studies are needed to explore a combination use of phage treatment with other techniques to fully remove *Salmonella* from sprouts food, and the resistance mechanisms in different *Salmonella* strains, including further analysis of the morphological, kinetic, and transcriptomic characteristics.

## CRediT authorship contribution statement

**Nan Xiang:** Conceptualization, Methodology, Investigation, Visualization, Writing – original draft. **Catherine W.Y. Wong:** Methodology, Writing – review & editing. **Xinbo Guo:** Methodology, Supervision. **Siyun Wang:** Supervision, Conceptualization, Writing – review & editing, Funding acquisition.

## Declaration of competing interest

The authors declare the following financial interests/personal relationships which may be considered as potential competing interests:

Siyun Wang reports financial support was provided by The University of British Columbia. Nan Xiang reports financial support was provided by South China University of Technology.

## Data Availability

Data will be made available on request.
